# Assessment of Median Nerve Mobility by Ultrasound Dynamic Imaging for Diagnosing Carpal Tunnel Syndrome

**DOI:** 10.1371/journal.pone.0147051

**Published:** 2016-01-14

**Authors:** Tai-Tzung Kuo, Ming-Ru Lee, Yin-Yin Liao, Jiann-Perng Chen, Yen-Wei Hsu, Chih-Kuang Yeh

**Affiliations:** 1 Department of Biomedical Engineering and Environmental Sciences, National Tsing Hua University, Hsinchu, Taiwan; 2 Department of Neurosurgery, Hsin-chu Mackay Memorial Hospital, Hsinchu, Taiwan; 3 Department of Biomedical Engineering, Hungkuang University, Taichung, Taiwan; 4 Department of Physical, Hsin-chu Mackay Memorial Hospital, Hsinchu, Taiwan; 5 Department of Neurology, Hsin-chu Mackay Memorial Hospital, Hsinchu, Taiwan; UCLA, UNITED STATES

## Abstract

Carpal tunnel syndrome (CTS) is the most common peripheral neuropathy and is characterized by median nerve entrapment at the wrist and the resulting median nerve dysfunction. CTS is diagnosed clinically as the gold standard and confirmed with nerve conduction studies (NCS). Complementing NCS, ultrasound imaging could provide additional anatomical information on pathological and motion changes of the median nerve. The purpose of this study was to estimate the transverse sliding patterns of the median nerve during finger movements by analyzing ultrasound dynamic images to distinguish between normal subjects and CTS patients. Transverse ultrasound images were acquired, and a speckle-tracking algorithm was used to determine the lateral displacements of the median nerve in radial-ulnar plane in B-mode images utilizing the multilevel block-sum pyramid algorithm and averaging. All of the averaged lateral displacements at separate acquisition times within a single flexion–extension cycle were accumulated to obtain the cumulative lateral displacements, which were curve-fitted with a second-order polynomial function. The fitted curve was regarded as the transverse sliding pattern of the median nerve. The *R*^2^ value, curvature, and amplitude of the fitted curves were computed to evaluate the goodness, variation and maximum value of the fit, respectively. Box plots, the receiver operating characteristic (ROC) curve, and a fuzzy c-means clustering algorithm were utilized for statistical analysis. The transverse sliding of the median nerve during finger movements was greater and had a steeper fitted curve in the normal subjects than in the patients with mild or severe CTS. The temporal changes in transverse sliding of the median nerve within the carpal tunnel were found to be correlated with the presence of CTS and its severity. The representative transverse sliding patterns of the median nerve during finger movements were demonstrated to be useful for quantitatively estimating median nerve dysfunction in CTS patients.

## Introduction

Carpal tunnel syndrome (CTS), was first described in 1854 by Paget [1], and is the most frequently encountered peripheral compression mononeuropathy, with a prevalence of 5% in the general population [2, 3]. The recent AAOS (American Academy of Orthopaedic Surgeons) Clinical Guidelines define CTS as a symptomatic compressive neuropathy of the median nerve at the level of the wrist characterized physiologically by evidence of increased pressure within the carpal tunnel and ultimately decreased function of the median nerve at that level [4]. Anatomically, the carpal tunnel is bounded by the fibrous transverse carpal ligament (TCL) on the volar side, and eight carpal bones on the dorsal side. Nine flexor tendons of the fingers and the median nerve pass through the carpal tunnel at the wrist level. The carpal tunnel can be grossly divided into a proximal part, approximately at the level of the pisiform bone, and a distal part, approximately at the level of the hook of the hamate bone. These bony structures could serve as anatomical landmarks for the quantitative analysis of the median nerve in imaging studies.

The diagnosis of CTS is usually based on constellations of clinical symptoms (e.g., numbness or tingling pain in the median nerve distribution of hand), provoking factors (e.g., sleep, or repetitive movement of the wrist), mitigating factors (e.g., shaking the hands, or changes in hand posture), and neurological findings (e.g., Tinel’s sign, Phalen’s sign, sensory impairment in the distribution of the median nerve, or thenar muscle atrophy) [5]. Neurophysiological studies of the median nerve—including nerve conduction studies (NCS) with or without electromyography—are currently used as the gold standard for the definitive diagnosis of CTS [6]. However, NCS are invasive, painful, time consuming and relatively expensive, and thus are poorly accepted by some patients. Moreover, it has been reported that the findings of electrodiagnostic testing (EDX) are normal in 16~34% of patients with clinically suggestive CTS [6–9]. Hence the disadvantages and diagnostic insufficiency of EDX have prompted researchers to investigate more convenient and effective tools for the definitive diagnosis of CTS. Complementary to NCS, magnetic resonance imaging (MRI) can provide visible anatomical information of the median nerve and neighboring flexor tendons across the carpal tunnel, such as compressive neuropathy, nerve edema, inflammation and even other subtle pathological causes of CTS (e.g. ganglion, haemangioma or bony deformity) and demonstrates superior ability of high imaging resolution [10–14]. With the advance of MR neurography which greatly improve the reliability of identification of the median nerve in images, some researchers have demonstrated the characterized transverse [15] and anterior-posterior movement of the median nerve within the carpal tunnel [16]. Moreover, Jarvik et al. concluded that the linear extent of the abnormal high intensity T2-weighted nerve signal on MRI and the median-ulnar sensory latency difference are both strong predictors of surgical benefit at 1 year, and there was a clear patient preference for MRI over electrodiagnostic testing (EDX) [14]. Nevertheless, the characteristics of high cost and the time consuming nature of scanning for MRI technique limit its popularity.

Ultrasound (US) imaging has advantages of simplicity, low cost, noninvasiveness, real-time capability and portability compared with traditional EDX. US imaging also offers high temporal and spatial resolutions, and can potentially provide dynamic anatomical information regarding local structures and kinesiology. Ultrasonography has been increasingly used during the past 2 decades to evaluate peripheral entrapment neuropathies such as CTS and tardy ulnar palsy, and the preliminary results have been promising [7, 17–31]. Most of these studies have focused on investigating morphological changes of the median nerve by measuring the cross-sectional area (CSA) and/or flattening ratio (FR) of the median nerve in the carpal tunnel (either the proximal or distal part), and changes in the thickness of the flexor retinaculum in transverse US images [17, 20, 32]. Some published reports have suggested that the CSA of the median nerve at the inlet of carpal tunnel represents the most effective diagnostic index (at the level of scaphoid-pisiform bone), with measuring sensitivities and specificities of 82~94% and 65~97%, respectively [18, 24, 27, 30]. Nevertheless, the wide variations of the sensitivity and specificity reported in the literature, which could be due to variations in the characteristics of CTS patients [e.g., race, body weight, body mass index (BMI), or wrist circumference], non-standardized measuring protocols, or inter-rater deviations, have prevented meaningful analysis of US as either a screening or confirmatory tool in the diagnosis of CTS [9]. Another crucial analytic limitation is that previous investigators have focused on morphological differences of the median nerve between normal controls and CTS patients, which does not allow estimation of the residual functional reserve of the median nerve in the affected patients compared with current standard NCS examinations.

The underlying pathophysiology of CTS has been copiously reported regarding median nerve dysfunction. The median nerve is known to be a flexible and mobile nerve structure that stretches and translates in response to changes in the motion of the adjacent flexor digital tendons when the fingers move in flexion and extension [33]. The median nerve is subject to being stretched or compressed against the TCL by constricted flexor digital tendons within the carpal tunnel during finger movements. Hence, the motion of the median nerve during wrist or finger movements could closely reflect the pathogenesis and/or pathomechanisms of CTS. Longitudinal excursion [34–39] and transverse sliding [40] of the normal median nerve during wrist and finger movements have been reported, and the authors of these preliminary studies proposed that the longitudinal excursion and transverse nerve sliding were reduced in CTS patients relative to normal subjects, combined with restriction of median nerve mobility during different finger movements as well as greater deformation of the median nerve [41–46]. These findings indicate that CTS is substantially a disorder involving restriction of the median nerve motion resulting from a decrease in the available functional space within the carpal tunnel and consequently increased carpal tunnel pressure. Hence, evaluating of median nerve mobility within the carpal tunnel could offer new insights into the pathomechanisms underlying CTS and for definitively diagnosing this condition.

Difficulties associated with diagnosing CTS based on motion of the median nerve include the likelihood that such motion is non uniform, and influenced by local anatomical, mechanical, and vasculature features and the movement mode of the flexor tendons. Altering the median nerve displacements in either the palmar-dorsal or radial-ulnar direction via active differential finger movements has rarely been reported in literature [33–37, 39–46]. Although previous studies have recorded changes in median nerve mobility longitudinally or transversely in normal subjects and CTS patients, no definitive patterns of the median nerve motions are available for use in current clinical examinations of CTS. Hence, the aims of this study were (1) to characterize the transverse sliding patterns of the median nerve during active finger flexion and extension movements by analyzing US B-mode dynamic images, (2) to determine variations in the patterns there in order to distinguish healthy subjects from CTS patients, and (3) to identify any correlation between the NCS severity and the median nerve mobility.

## Materials and Methods

### Participant data collection including NCS as a reference

The research protocol used in this study was approved by the Institutional Review Board of Hsin-chu Mackay Memorial Hospital (IRB #12MMHIS195), and all participants signed written informed consents. This study recruited 32 normal wrists, 26 wrists with mild CTS, and 14 wrists with severe CTS—as confirmed by electrophysiological NCS—during 2012 and 2013. Those who had a past history of cervical radiculopathy, diabetes mellitus or glucose intolerance, hypothyroidism, rheumatoid arthritis, gout, hemodialysis, wrist osteoarthritis, sarcoidosis, amyloidosis or previous traumatic insult to the affected wrist were excluded.

NCS were performed in all participants as the gold-standard reference for the diagnosis of CTS. Various parameter estimates have been proposed in the literature, and this study adopted the clinical standard confirmatory protocol based on the American Association of Electrodiagnostic Medicine summary statement [47]. That is, a diagnosis of CTS was established if a subject had a sensory conduction velocity (SCV) of <40 m/s, a distal sensory latency (DSL) of >2.5 ms, a distal motor latency (DML) of >4.0 ms, or a waveform amplitude of less than 8 mV. Furthermore, using the data from the NCS, we defined mild CTS as an SCV of 30~40 m/s, a DSL of 3.0~4.4 ms, or a DML of 4.4~6.4 ms as, while severe CTS was defined as a SCV of <30 m/s, a DSL of >4.4 ms, a DML of >6.4 ms or an undetectable waveform [48, 49].

### US scanning protocol

**Fig 1:**
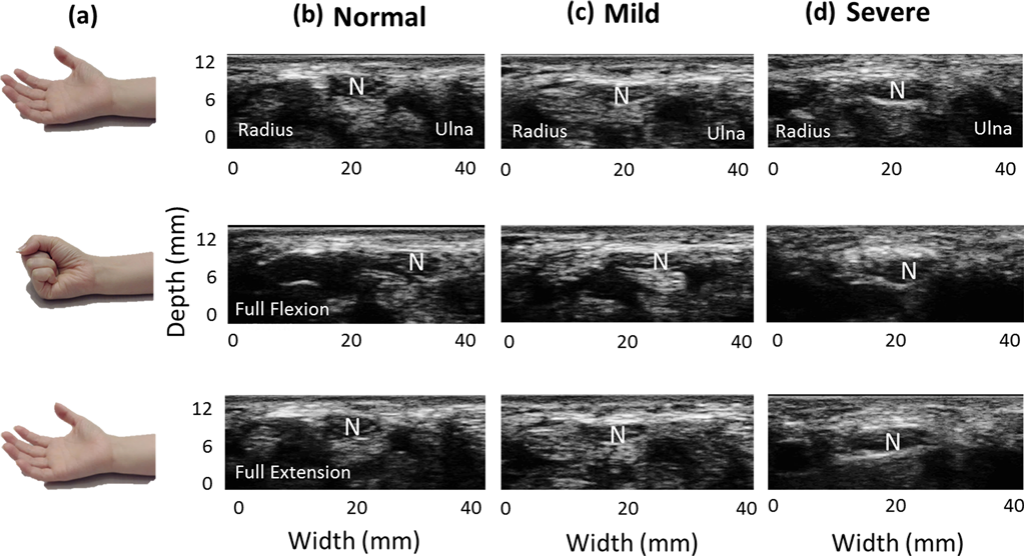
Ultrasound scanning. While the fingers performed active flexion and extension movements (a), the transverse sliding motion of the median nerve within the carpal tunnel was clearly identified in dynamic B-mode images. (b), (c) & (d) Representative US images obtained from normal subjects and mild- and severe- CTS patients, respectively. Note that the amount of transverse sliding of the median nerve varied with the severity of CTS. N indicates the median nerve. Normal, Mild, and Severe indicate normal subjects, mild-CTS and severe-CTS patients.

A commercial US scanner (Model t3000, Terason, MA) with a 10-MHz linear array probe (Model 12L5A, Terason) was used for clinical image acquisition in this study. The frame rate was set as 25 fps, and the parameter settings of the scanner such as the optimal gain, uniform time gain compensation, and focus depth were identical in all subject examinations. Cross-sectional images of the carpal tunnel were acquired by an experienced musculoskeletal physician who had performed clinical US image acquisition for more than 10 years. The subjects were positioned with the forearm supine on a soft flat plane (e.g., a pillow) and the wrist maintained in a neutral position. The transducer was placed transversely at the level of the distal wrist crease and perpendicular to the long axis of the forearm, just at the inlet of the carpal tunnel. The participants were requested to perform neutral extension of their fingers initially, followed by full flexion (clenched-fist posture) and then back to finger extension (open-palm posture)—that is active finger movements in flexion–extension cycles [Fig 1(A)]—, while 69~70 frames of B-mode scan images were acquired at intervals of about 3 seconds [Fig 1(B)–1(D)]. Active finger movements in flexion and extension induce passive displacements of the median nerve, and the transverse sliding of the median nerve within the carpal tunnel during finger movements can be clearly observed in US dynamic imaging. Furthermore, as shown in Fig 1(B), during one finger flexion–extension cycle, the median nerve presented a neutral position initially, and then moved toward the ulnar side transversely when the fingers were at full flexion, and finally returned back to the radial side transversely when the fingers were extended. The ovoid median nerve was identified in B-mode image, and the outermost hyperechoic rim of the median nerve was outlined manually for further measurements of the transverse displacements.

### Estimation of the pattern of median nerve motion

The transverse displacement of the median nerve in the radial-ulnar plane within the carpal tunnel was measured by applying a speckle-tracking algorithm to sequential US dynamic images. This study utilized a multilevel block-matching and block-sum pyramid (BSP) integrated algorithm (referred to here as the multilevel BSP algorithm) for speckle tracking between sequential US B-mode images. The multilevel BSP algorithm had been demonstrated to achieve excellent computational performance for two-dimensional speckle tracking in B-mode images [50–52].

The multilevel BSP algorithm is comprises of a matching process and a searching process. The matching process was based on a BSP algorithm, which significantly reduces the computational complexity while maintaining the same accuracy as the conventional sum-of-absolute-differences approach. The searching process, on the other hand, was based on a multilevel search strategy involving comparing matching blocks on a level-by-level basis rather than the full-search strategy used by most conventional tracking methods. This improved strategy allowed real-time (or near-real-time) implementation of motion estimation in US imaging. We chose a region of 32 pixels by 32 pixels (15.56 pixels/mm) as a matching block in the reference image (i.e., original image) and compared this to each test block of the same size in the search region of the comparison image. The search block comprised 21 pixels by 21 pixels, which ensured that it covered at least 10 independent speckles and provided acceptable speckle-tracking results. The displacement was estimated as the position difference of the best-matched pixel between the test block in the comparison image (i.e., the *i*th frame) and the matching block in the fourth previous image [i.e., the matching (*i***–**4)th frame]. This process continued until the displacements for all pixels in the center of the matching block in the original image were acquired.

**Fig 2:**
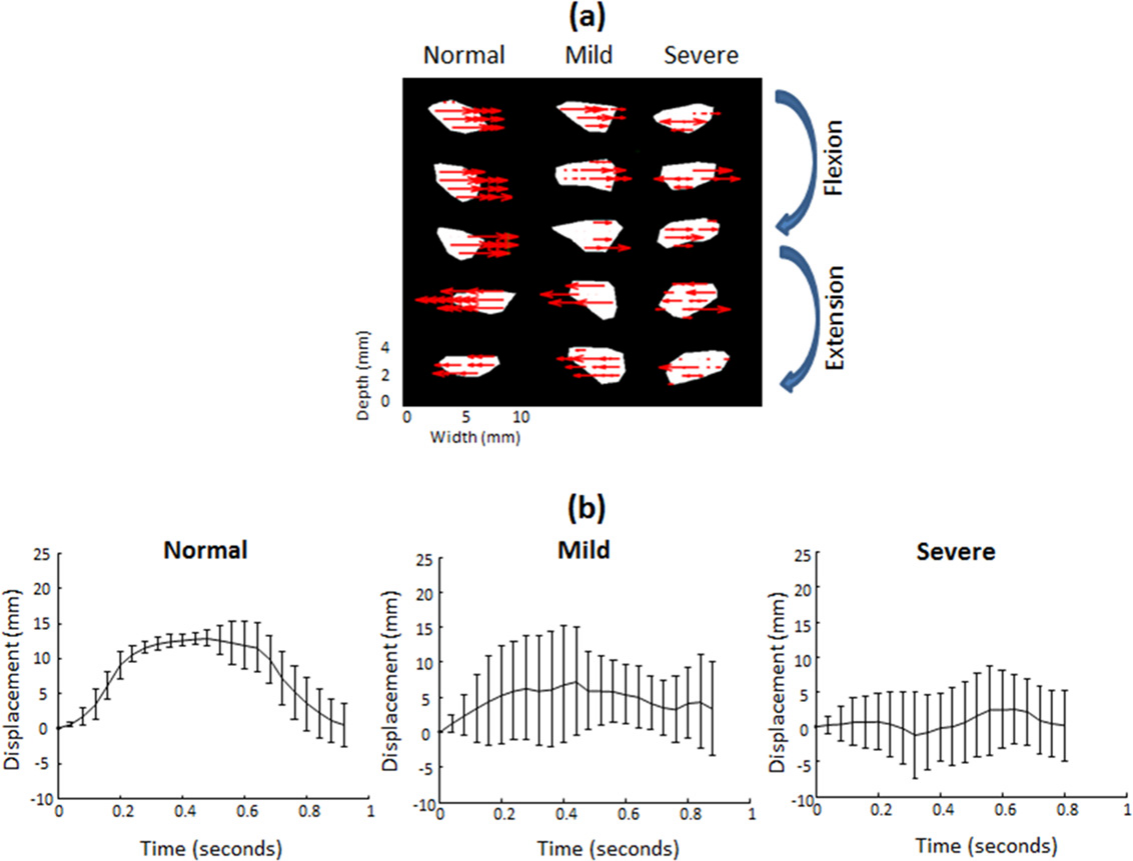
Estimation of the pattern of median nerve motion. During finger flexion and extension movements in normal subjects and mild-CTS and severe-CTS subjects, the median nerve showed non uniform transverse sliding motion over the ulnar-radial plane. (a) Red arrows indicate the direction of lateral displacements of representative pixels within the median nerve as calculated using a speckle-tracking algorithm. (b) Cumulative average lateral displacements (mean and SD values) of the median nerve at different acquisition times during one finger flexion–extension cycle, indicating marked variations of the motion patterns between normal subjects and mild-CTS and severe-CTS patients. Normal, Mild, and Severe indicate normal subjects, mild-CTS and severe-CTS patients.

In this study we focused on the median nerve as the target region of interest in cross-sectional US images, and used the multilevel BSP algorithm to calculate the lateral displacement of each pixel within the median nerve boundary between sequential images, as shown in Fig 2(A). Differential movements of the fingers in flexion or extension meant that the direction and amount of lateral displacement of the median nerve varied between normal subjects and patients with mild or severe CTS. The transverse sliding motions of the median nerve toward the ulnar and radial directions during the finger flexion and extension movements were defined as positive and negative displacements, respectively [Fig 2(A)]. Note that the lateral displacement of the median nerve evident in sequential B-mode images corresponded to the averaged transverse displacement of all pixels contained within the median nerve boundary. All of the average lateral displacements at different acquisition time points acquired in an entire finger flexion–extension cycle were then accumulated to obtain cumulative lateral displacements, as shown in Fig 2(B), which illustrates marked variation of the motion patterns among normal subjects and patients with mild or severe CTS.

In theory, the transverse motion of the median nerve over the ulnar-radial axis involves an identical traversed distance but in opposite directions during finger flexion and extension as the participant performs standardized repetitive finger movements. We therefore assumed that the resultant cumulative lateral displacements versus acquisition time (i.e., temporally cumulative lateral displacements) over one cycle of finger movements would appear symmetric for the transverse sliding of the median nerve, and that the temporally cumulative lateral displacements can be curve-fitted by a second-order polynomial function. Note that the fitted curve was regarded as the transverse sliding pattern of the median nerve within the carpal tunnel during finger flexion and extension movements.

### Statistical analysis

To differentiate normal subjects from CTS patients, the estimated transverse sliding patterns of different subgroups were compared using three statistical parameters: *R*^2^, curvature and amplitude. Since the fitted curves were derived from the temporally cumulative lateral displacements by a second-order polynomial function, the *R*^2^, curvature, and amplitude estimates represent the goodness of fit to the temporal cumulative lateral displacement of the median nerve, the variation of the fitted curve, and the maximum value of the fit, respectively.

Physiologically, the *R*^2^ estimate could account for the transverse sliding ability or function of the median nerve in response to active finger movements, and we could regard the curvature and amplitude estimates as representing the functional compliance or elasticity of the median nerve, and the maximal transverse sliding displacement of the median nerve or the conceptually spatial pressure within the carpal tunnel in response to active finger movements, respectively.

The three parameters were then compared statistically between the different subgroups, with the data expressed as mean±standard deviation values. Student’s *t*-test was used to assess the statistical significance of differences in the values of each parameter between normal subjects and CTS patients. An overall probability value of less than 0.05 was assumed to be indicative of statistical significance. The values of the three variables were presented using box plots to facilitate the visual differentiation of normal subjects and CTS patients. The receiver operating characteristic (ROC) curve was utilized to evaluate the diagnostic performances of each estimate and the overall composite estimate in discrimination between normal subjects and CTS patients. ROC curve is a graphical plot that statistically estimates the quality or performance of a binary classifier system as its discrimination cutoff is varied. ROC analysis is a useful tool for evaluating the performance of diagnostic tests and more generally for evaluating the accuracy of a statistical model (e.g., logistic regression, linear discriminant analysis) that classified subjects into 1 of 2 categories, diseased or nondiseased. The area under the ROC curve (AUC) is a measure of how well a parameter can distinguish between two diagnostic groups (diseased/nondiseased). The three parameters was then inputted into a fuzzy c-means (FCM) algorithm which is an unsupervised clustering method for dividing a group of data points into two clusters/classes for representing the correlations between the different parameter attributes.

## Results

US dynamic imaging readily demonstrated that the normal median nerve moves in the ulnar direction during finger full flexion and backward in the radial direction during finger extension. Moreover, the median nerve displacement in the radial-ulnar plane was reduced in CTS patients compared with normal subjects [Fig 1(B)]. Consequently, estimating changes in such transverse sliding of the median nerve in the carpal tunnel would provide better insight into the specific causative factors in individual patients.

The transverse sliding of median nerves during finger flexion and extension was greater in the normal subjects than in the patients with mild or severe CTS. In addition, a smaller amount of median nerve motion was correlated with more-severe NCS, which implied that the decreasing median nerve mobility was correlated with the reduced median nerve conduction velocity.

**Fig 3:**
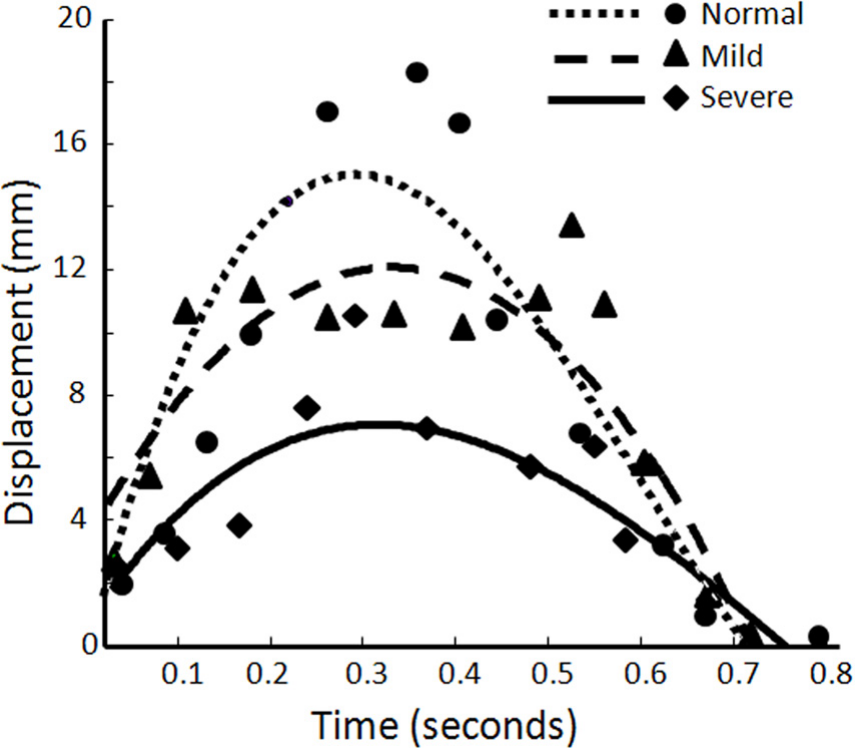
Transverse sliding patterns of the median nerve. Representative fitted curves indicating the various transverse sliding patterns of the median nerve during fingers flexion and extension movements among a normal subject, a mild-CTS patient, and a severe-CTS patient. The dots, triangles, and diamonds represent the cumulative lateral displacements at different acquisition times and the intersecting lines indicate the fitted curves for the different subgroups, respectively. Normal, Mild, and Severe indicate normal subjects, mild-CTS and severe-CTS patients.

The transverse sliding patterns of the median nerve during finger flexion and extension movements for a normal subject, a mild-CTS patient, and a severe-CTS patient could be expressed as shown in Fig 3. The slopes of the fitted curve in both the increasing and decreasing segments were greater in normal subjects than in patients with mild or severe CTS. Because the normal median nerve had a larger and more regular transverse sliding motion than the CTS-affected median nerve, its temporally cumulative lateral displacement fitted curve appeared to increase stably and then decrease stably, resulting in the effective fitted uppermost dashed line shown in Fig 3. On the other hand, the fitted curve for the patients with mild or severe CTS appeared to not fit the temporally cumulative lateral displacements of the median nerve, reflecting the irregular and impaired transverse sliding of the CTS-affected median nerve in the carpal tunnel during active finger movements. In addition, the transverse-sliding-pattern curve of the median nerve was steeper in mild-CTS patients than in severe-CTS patients.

**Fig 4:**
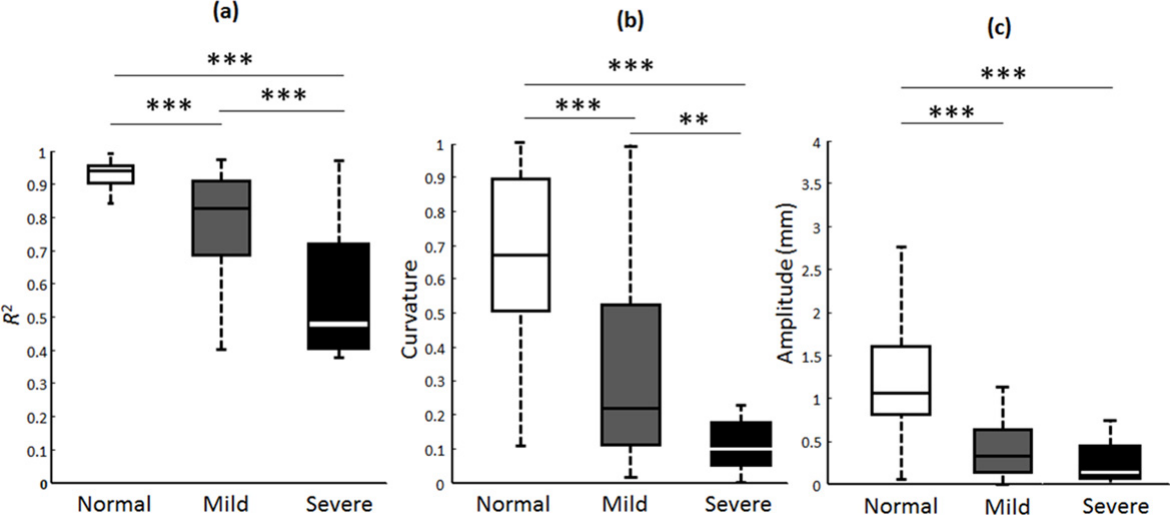
Box plots analysis for the *R*^2^, amplitude, and curvature estimates. The calculated distributions of *R*^2^, amplitude, and curvature estimates of the fitted curves for the normal subjects, mild-CTS and severe-CTS patients were presented. The bisecting line, box boundaries, and whiskers indicate the median value, 25^th^ to 75^th^ percentiles, and the estimated data range, respectively. Two and three asterisks indicate *p* < 0.01 and *p* < 0.001, respectively. Normal, Mild, and Severe indicate normal subjects, mild-CTS and severe-CTS patients.

The *R*^2^, curvature and amplitude estimates of the fitted curves were 0.94±0.02, 0.69±0.28, and 1.27±0.62, respectively, for the normal subjects; 0.77±0.15, 0.25±0.23, and 0.57±0.42 for the mild-CTS patients; and 0.56±0.19, 0.12±0.11, and 0.35±0.31 for the severe-CTS patients. Using box plots as shown in Fig 4, compared to the CTS patients, the normal subjects had significantly higher *R*^2^, curvature and amplitude estimates of the fitted curves. The *R*^2^, curvature, or amplitude estimate provided an excellent ability to distinguish normal subjects from CTS patients, regardless of their disease severity (*p* < 0.001). Furthermore, the curvature and *R*^2^ estimates showed good-to-excellent power in differentiating between mild- and severe-CTS patients (*p* < 0.01 and *p* < 0.001, respectively), whereas the amplitude estimate did not differ significantly between patients with mild and severe CTS.

**Fig 5:**
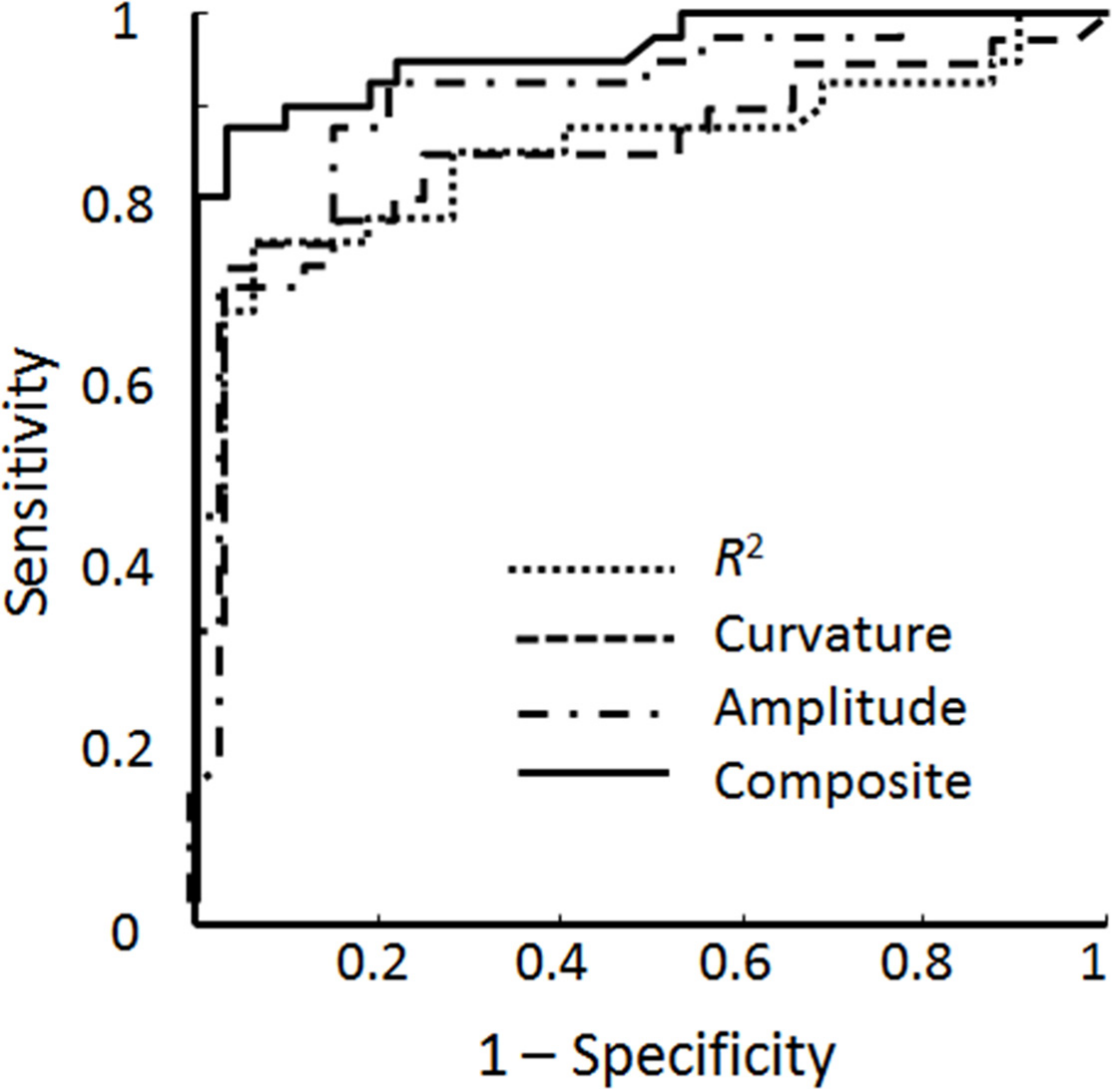
Receiver operating characteristic (ROC) curves analysis. The diagnostic performances of the *R*^2^, curvature, and amplitude estimates alone and of the overall composite estimates in distinguishing normal subjects from CTS patients were demonstrated.

ROC curve analysis was used to determine the performance metrics—including sensitivity, accuracy, and specificity—of the three individual parameters, as shown in Fig 5. Table 1 lists the specificity, sensitivity, accuracy, and area under the ROC curve (AUC) of the different parameters assessed by ROC curves. The computed AUCs of the *R*^2^, amplitude, and curvature estimates were 0.851, 0.899, and 0.857, respectively, and yielded accuracies of 83.3%, 86.1%, and 83.3%, as presented in Table 1. This indicates that the amplitude estimate was the best performer of the three estimates, with a sensitivity of 87.5% and a specificity of 84.4%. The overall composite analysis for augmenting the diagnostic efficacy estimation demonstrated a better result, with an accuracy of 91.7%, specificity of 96.9%, sensitivity of 87.5%, and an AUC of 0.96, which indicates that combining the *R*^2^, curvature, and amplitude estimates can greatly improve the efficacy in diagnosing CTS compared to applying only one parameter attribute.

**Table 1:** Diagnostic performance of different parameters analyzed with ROC curves.

	Parameter			
Performance metric	*R*^2^	Curvature	Amplitude	Composite
Specificity	93.8%	93.8%	84.4%	96.9%
Sensitivity	75%	75%	87.5%	87.5%
Accuracy	83.3%	83.3%	86.1%	91.7%
AUC	0.851	0.857	0.899	0.96

**Fig 6:**
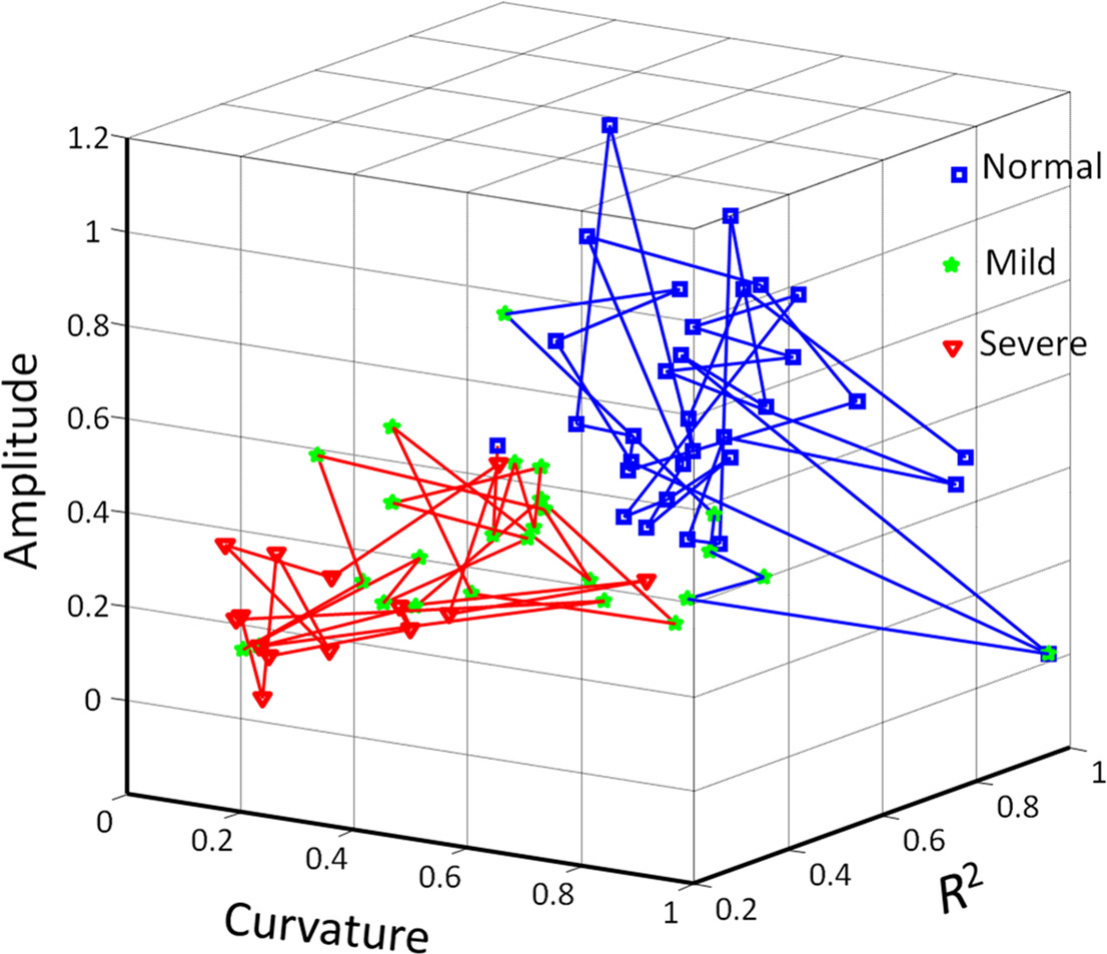
Three-dimensional FCM clustering analysis. Distinguishing normal subjects from CTS patients by combining three parameters was shown. Each symbol represents an individual subject. Blue and red lines indicate estimated normal and CTS clusters, respectively. Normal, Mild, and Severe indicate normal subjects, mild-CTS and severe-CTS patients.

Since weaker correlations among different parameter sets implied that the discrimination performance could be improved, we tested the efficacy of combining the three parameters in the FCM clustering algorithm in distinguishing between normal subjects and CTS patients. A representative illustration is presented in Fig 6. The data points in the figure are divided into two clusters representing normal subjects and CTS patients. Based on the numbers of data points, the high diagnostic efficacy when using FCM clustering was indicated by an accuracy of 90.3%, a specificity of 96.9%, and a sensitivity of 85%. Compared to using each respective parameter alone, combining the *R*^2^, amplitude and curvature estimates can clearly improve the diagnostic efficacy.

## Discussion

This study has proposed a novel model for the quantitative measurement of median nerve dysfunction, which is crucial for understanding the pathomechanisms underlying CTS and for diagnosing this condition. Several researchers have investigated changes in the motion of the median nerve and even the individual finger flexor tendons within the carpal tunnel using US for evaluating the kinematics within the carpal tunnel, in an attempt to understand the pathomechanisms underlying CTS [33, 41–46, 53]. Yoshii et al. reported the displacement and deformation of the median nerve during different finger movements as determined using transverse US imaging [33], and van Doesburg et al. reported changes in the motion pattern of the median nerve and some of the flexor tendons [46]. Nevertheless, these preliminary studies did not quantitatively analyze mobility dysfunction of the median nerve. Although motion of the median nerve exhibits a non uniform pattern dependent on the local anatomical and biomechanical properties and the specific finger or wrist movements involved, we have demonstrated in this study that the transverse sliding of the median nerve within the carpal tunnel during standardized active finger movements can be substantially converted into analyzable estimates for differentiating between normal subjects and CTS patients.

In B-mode images, the normal median nerve exhibited greater transverse sliding displacement during active finger flexion and extension movements than that found in patients with mild or severe CTS. In addition, a smaller amount of median nerve motion was associated with more severe NCS. This implies that impairment of median nerve mobility is correlated with impaired median nerve conduction velocity and the subsequent median nerve dysfunction. Moreover, considering one cycle of finger flexion and extension movements, we assume that the normal median nerve always moves transversely over the ulnar-radial axis in a to-and-fro manner with an identical traversed distance and stops at the original neutral position. Hence, the temporally cumulative lateral displacements of the median nerve during one cycle of finger movements will theoretically appear as a symmetric distribution of transverse sliding (Fig 2). We can thus draw the fitted curve using a second order polynomial function, which ideally presents a symmetric parabolic curve.

The fitted curves of temporally cumulative lateral displacements were regarded as the transverse sliding patterns of the median nerve within the carpal tunnel during finger flexion and extension movements, which is a key point in this study. It is reasonable that the median nerve moved transversely over a larger distance and with more regularity in the patients with milder CTS, and the corresponsive fitted curve would more likely present as the standard parabolic curve exhibited by normal subjects. Therefore, the fitted curve of temporally cumulative lateral displacements would become more flattened and irregular (i.e., more asymmetric) as the severity of CTS affecting the median nerve increases (Fig 3). Nevertheless, the actual transverse-sliding-pattern curves of the median nerves over a single cycle of finger movements did not appear as symmetric as a parabolic curve, which might have been due to the bias in the acquisition of US images, such as the non standardized speed and/or force during finger movements; however this should hardly affect the subsequent statistical analyses since the processed fitting curves were acceptable.

The box plots of Fig 4 indicate that the amplitude estimate could not be used to differentiate mild- and severe-CTS patients, whereas the results of the ROC curve analysis in Fig 5 indicate that the amplitude estimate provided the best diagnostic accuracy of the three parameters. Hence, the disequilibrium of and absence of correlation between different parameter sets in the box plots and ROC curve analyses indicated to us that the various parameter attributes cannot depict the representative transverse sliding pattern of the median nerve within the carpal tunnel during finger movements and that the discrimination performance needs to be further improved. Therefore, the FCM algorithm was adopted to verify the correlation and the discrimination power of the three parameter estimates. The good diagnostic performance of FCM clustering was confirmed by a sensitivity of 85%, a specificity of 96.9% and an accuracy of 90.3%; these metrics are comparable with the results of the overall composite ROC curve analysis. A review of the literature revealed that a recent large series of meta-analysis research found the composite pooled sensitivity and specificity of US imaging for the diagnosis of CTS to be 77.6% [95% confidence interval (CI) = 71.6%–83.6%] and 86.8% (95% CI = 78.9%–94.8%), whereas the sensitivity and specificity when using EDX as the reference standard were 80.2% and 78.7% [9]. In a recent literature, Fowler et al. reported that using a validated clinical tool (CTS-6) as the reference standard, US had a sensitivity of 89% and a specificity of 90%, and NCS had a sensitivity of 89% and a specificity of 80% in the diagnosis of CTS [54]. In our study, the diagnostic performance of estimated sensitivity and specificity of our model using NCS as gold-standard reference is comparable with those in publication by Fowler et al. Moreover, in their study, they chose an a priori cutoff ≥10 mm^2^ at the inlet to the carpal tunnel as the cutoff for a positive US examination, whereas we attempted to ameliorate the diagnostic performance of US by quantitatively assessing the median nerve mobility across the carpal tunnel during finger movements using a US dynamic imaging technique for diagnosing CTS. As a preliminary study, we adopted the currently accepted reference standard (NCS) instead of clinical examination as our gold-standard reference for statistical analysis. We can therefore postulate that the model proposed in this study provides a comprehensive representation of the median nerve dynamics within the carpal tunnel for assessing the functional status of the median nerve in the diagnosis of CTS, and provides satisfactory results based on current standard neural electrophysiological studies.

This study was subject to some limitations. First, the inability to discriminate some mild- and severe-CTS patients using FCM clustering method may be related to the relative smallness of the sample (32 normal subjects, 26 mild-CTS patients and 14 severe-CTS patients) used for characterizing the CTS severity. Second, when the participants were performing the cycle from finger extension to a clenched fist during each 3-second acquisition time, the palmar muscle contractile force was not uniformly controlled and also could not be measured objectively, which could have influenced the analysis of the pattern of median nerve motion. Third, a speckle-tracking algorithm was utilized to calculate the transverse displacement of the median nerve within the carpal tunnel over one cycle of finger movements in our study, but the presence of severe adhesion of the median nerve observed in severe CTS patients may have introduced some analysis errors that lead to bias. Fourth, the diverse characteristics of the included individuals, such as in their gender, body weight, height, BMI, and wrist circumference, was not allowed for in the statistical analyses, which might also have contributed further bias.

Some investigators had advocated that changes in biomechanical properties such as thickening and noninflammatory fibrosis of the subsynovial connective tissue (SSCT) within the carpal tunnel play an essential role of the pathophysiology of CTS [33, 42, 53, 55–62]. The SSCT loosely connects the finger flexor tendons, median nerve, and synovium, and serves as a sliding unit to reduce friction and protect the meshwork of the vasculature during tendon motion [63–65]. Thus, thickening and fibrosis of the SSCT might hinder motion of the median nerve and even of the finger flexor tendons within the carpal tunnel. Previous microtrauma insults caused by either mechanical damage [65] or ischemia-reperfusion injury [66] may significantly increase the fibroblast activity and density, since increased expression of transforming growth factor-β, collagen fibril size, vascular proliferation, and type III collagen have been noted in the SSCT in histological examination [61]. The above-described changes cause scarring and fibrosis around the median nerve and flexor tendons which in turn can tether the nerve [67]; this was assessed by the *R*^2^ estimate for the transverse sliding function of the median nerve in response to standardized finger movements in our study. On the other hand, the altered properties of the SSCT would not only secondarily contribute to increase the volume of the contents within the carpal tunnel, but also may decrease the tissue compliance and permeability of the vasculature, leading increased hydrostatic pressure, and this phenomenon was assessed by the curvature estimate in the present study. The detrimental effect of these alterations in turn predispose the nerve and SSCT to secondary injury and lead to the elevation of carpal tunnel pressure that is seen in CTS patients [61], which would hinder the amount of transverse sliding by the median nerve and was accounted for by the amplitude estimate in our study. Hence, evaluating median nerve mobility and kinematics within the carpal tunnel using our proposed US dynamic-imaging-based discrimination model would allow affected and unaffected subjects to be distinguished, and provides insight into the pathomechanisms underlying CTS.

## Conclusions

This study has proposed a novel model for quantitatively evaluating median nerve mobility in the radial-ulnar plane within the carpal tunnel during finger movements using a US dynamic imaging technique, with the aim of differentiating normal subjects from CTS patients and basically is useful for the computer-aided diagnosis (CAD) of the CTS. In present study, quantitative analysis for the median nerve mobility after the examination of normal subjects or CTS patients in a separate step (post-hoc computer based analysis) is still needed, and usually we can readily observe the restricted motion of the median nerve within the carpal tunnel in CTS patients during the US dynamic imaging examination. Given well-developed and mature modules, the processing formula proposed in our study can be integrated into the commercialized US machine software, and introducing the newly acquired model knowledge in the daily clinical routine will be anticipated. The preliminary results obtained in the present study are encouraging; future studies could integrate static features, such as altered CSA, FR, and wrist-to-forearm ratio self-normalization of the median nerve morphology, which have been demonstrated to be valuable in the diagnosis of CTS [17–32, 68–70], and also dynamic features of the median nerve motion might be helpful in diagnosing of CTS and determining the CTS severity. In addition, measurements of the mechanical strain or stress using US (in US strain imaging) at different neural sites of the median nerve in the carpal tunnel could elucidate the elasticity function of the median nerve for the US-based diagnosis of CTS.
